# Supplementary material for: Common genetic variation associated with Mendelian disease severity revealed through cryptic phenotype analysis
Source: Nat Commun. 2022 Jun 27;13:3675. doi: 10.1038/s41467-022-31030-y (PMC9237040; doi:10.1038/s41467-022-31030-y)
Supplement: Supplementary file 3 — Description of Additional Supplementary Files [file 41467_2022_31030_MOESM3_ESM.pdf]

Description of Additional Supplementary Files

Supplementary Data 1: Tab-delimited text file containing the 50 Mendelian diseases analyzed in this study. The file provides the following information for each disease: unique ID (created for the purpose of this study), name, inheritance pattern(s), corresponding ICD10 code(s) (in both ICD10-CM and ICD10-UKBB format), and prevalence in the UCSF and UKBB datasets.

Supplementary Data 2: Tab-delimited text file containing the HPO-ICD10 alignments generated for this study. The file provides the following information for each HPO-ICD10 alignment: unique ID (created for this study), aligned HPO identification number(s), HPO string(s), HPO synonym(s), aligned ICD10-CM code(s), and aligned ICD10-UKBB code(s).

Supplementary Data 3: Tab-delimited text file containing the disease-HPO symptom alignments used in this study (prior to any downstream filtering).

Supplementary Data 4: Tab-delimited text file containing the increase in cryptic phenotype severity observed among diagnosed cases for the 38 Mendelian diseases whose phenotype models converged in both datasets. This information is provided for the UCSF model applied to the UCSF dataset (UCSF/UCSF), the UKBB model applied to the UCSF dataset (UCSF/UKBB), and the UKBB model applied to the UKBB dataset (UKBB/UKBB). 95% confidence intervals and bootstrapped *P*-values are also provided. The next five columns contain Boolean values indicating whether each disease survived the various replication filters. The final column provides the R^2-values for the cryptic phenotypes produced by the UCSF and UKBB models when applied to the subjects in the UCSF dataset. This data was used to generate **Figures 2d**, **2e**, and **2f**.

Supplementary Data 5: Tab-delimited text file containing the final HPO/ICD10 symptoms used for model inference for the 10 Mendelian diseases that survived all filtering steps. See column headings for details. Note, the columns `Annotated HPO Terms UCSF` and `Annotated HPO Terms UKBB` may contain a subset of the annotations provided in Supplementary Data File 2. Additional details are provided in the Supplementary Methods.

Supplementary Data 6: Tab-delimited text-file providing the Mendelian disease pathogenic variants used in the analyses presented in this manuscript. All alleles and positions correspond to the GRCh38 reference genome.

Supplementary Data 7: Tab-delimited text-file providing predictor effect sizes for the regression model used to produce the polygenic scores for A1ATD. Please see the `LDAK Toolkit` (https://dougspeed.com/profile-scores/) for additional details.

Supplementary Data 8: Tab-delimited text-file providing predictor effect sizes for the regression model used to produce the polygenic scores for AS. Please see the `LDAK Toolkit` (https://dougspeed.com/profile-scores/) for additional details.

Supplementary Data 9: Tab-delimited text-file providing predictor effect sizes for the regression model used to produce the polygenic scores for ADPKD. Please see the `LDAK Toolkit` (https://dougspeed.com/profile-scores/) for additional details.
