# Supplementary material for: Common genetic variation associated with Mendelian disease severity revealed through cryptic phenotype analysis
Source: Nat Commun. 2022 Jun 27;13:3675. doi: 10.1038/s41467-022-31030-y (PMC9237040; doi:10.1038/s41467-022-31030-y)
Supplement: Supplementary file 5 — Reporting Summary [file 41467_2022_31030_MOESM5_ESM.pdf]

nature portfolio

Corresponding author(s): David R. Blair; Joseph T. Shieh

Last updated by author(s): April 29th 2022

Reporting Summary

Nature Portfolio wishes to improve the reproducibility of the work that we publish. This form provides structure for consistency and transparency in reporting. For further information on Nature Portfolio policies, see our Editorial Policies and the Editorial Policy Checklist.

Statistics

For all statistical analyses, confirm that the following items are present in the figure legend, table legend, main text, or Methods section.

n/a	Confirmed	
☐	☒	The exact sample size (*n*) for each experimental group/condition, given as a discrete number and unit of measurement
☐	☒	A statement on whether measurements were taken from distinct samples or whether the same sample was measured repeatedly
☐	☒	The statistical test(s) used AND whether they are one- or two-sided<br>*Only common tests should be described solely by name; describe more complex techniques in the Methods section.*
☐	☒	A description of all covariates tested
☐	☒	A description of any assumptions or corrections, such as tests of normality and adjustment for multiple comparisons
☐	☒	A full description of the statistical parameters including central tendency (e.g. means) or other basic estimates (e.g. regression coefficient) AND variation (e.g. standard deviation) or associated estimates of uncertainty (e.g. confidence intervals)
☐	☒	For null hypothesis testing, the test statistic (e.g. *F*, *t*, *r*) with confidence intervals, effect sizes, degrees of freedom and *P* value noted<br>*Give P values as exact values whenever suitable.*
☒	☐	For Bayesian analysis, information on the choice of priors and Markov chain Monte Carlo settings
☒	☐	For hierarchical and complex designs, identification of the appropriate level for tests and full reporting of outcomes
☒	☐	Estimates of effect sizes (e.g. Cohen's *d*, Pearson's *r*), indicating how they were calculated

Our web collection on statistics for biologists contains articles on many of the points above.

Software and code

Policy information about availability of computer code

Data collection	No software was specifically written for data collection, as all data was previously collected by other researchers. Any scripts written for data processing used Python (version 3.6.13), it's associated standard libraries, NumPy (version 1.19.5), and pandas (version 1.2.1). Additional information regarding the software developed for this study is described below.
Data analysis	We have deposited the software developed for the analyses described in this manuscript on Github as follows:<br>1) vLPI: software package for cryptic phenotype inference (https://github.com/daverblair/vlpi)<br>2) CrypticPhenoImpute: software package for cryptic phenotype replication (https://github.com/daverblair/CrypticPhenoImpute)<br>3) CrypticPhenotypeAnalysisScripts: collection of scripts used to perform the analyses described in this manuscript (https://github.com/daverblair/CrypticPhenotypeAnalysisScripts)<br>Note, this software is publicly available with a permissive (MIT) license.<br><br>Below is a list of additional, third-party, open-source software used to complete the analyses in this study:<br>1) Python (version 3.6.13)<br>2) NumPy (version 1.19.5)<br>3) SciPy (version 1.5.3)<br>4) statsmodels (version 0.13.1)<br>5) scikit-learn (version 0.22.1)<br>6) pandas (version 1.2.1)<br>7) plink2 (version 2.00a3LM)<br>8) bcftools (version 1.12)<br>9) FUMA (version 1.3.6a)<br>10) Ensembl Variant Effect Predictor (version 103.1)<br>11) LOFTEE plug-in<br>12) LDAK Toolkit (version 5.1)

13) lifelines (version 0.26.0)
Citations to the above third-party software can be found in the main text.

For manuscripts utilizing custom algorithms or software that are central to the research but not yet described in published literature, software must be made available to editors and reviewers. We strongly encourage code deposition in a community repository (e.g. GitHub). See the Nature Portfolio guidelines for submitting code & software for further information.

Data

Policy information about availability of data

All manuscripts must include a data availability statement. This statement should provide the following information, where applicable:

- Accession codes, unique identifiers, or web links for publicly available datasets
- A description of any restrictions on data availability
- For clinical datasets or third party data, please ensure that the statement adheres to our policy

The clinical and genetic datasets used in the analyses presented in this manuscript cannot be shared directly with third parties, as both have specific provisions against open data sharing outside of their usual application processes. Information regarding third party access to the UCSF De-Identified Clinical Data Warehouse can be found through UCSF Data Resources: https://data.ucsf.edu/cdrp/research, and the application process for access to the UK Biobank is outlined on their website: https://www.ukbiobank.ac.uk/register-apply.

Datasets that were generated to conduct the analyses described in this manuscript are provided as Supplementary Data Files 1-9. The summary statistics for the cryptic phenotype genome-wide association studies were submitted to the GWAS catalog: https://www.ebi.ac.uk/gwas/ (accession IDs: GCST90101825, GCST90101826, GCST90101827, GCST90101828, and GCST90101829 for A1ATD, HHT, MFS, AS, and ADPKD respectively). The cryptic phenotypes for A1ATD, HHT, MFS, AS, and ADPKD were also returned to the UK Biobank (Application ID 53312).

The following, freely available, third-party datasets were used in the analyses presented in this study:
1) ClinVar: https://www.ncbi.nlm.nih.gov/clinvar/
2) Human Disease Ontology: https://obofoundry.org/ontology/doid.html
3) Humaun Phenotype Ontology: https://hpo.jax.org/app/
4) LOOM HPO-to-ICD9 and HPO-to-ICD10 Alignments: https://bioportal.bioontology.org/
5) UMLS Metathesaurus: https://www.nlm.nih.gov/research/umls/knowledge_sources/metathesaurus/index.html
6) US Edition of SNOMED: File tls_Icd10cmHumanReadableMap US1000124 20190301.tsv at https://www.nlm.nih.gov/healthit/snomedct/us_edition.html
7) ICD9-to-ICD10 map from the National Bureau for Economics Research: https://www.nber.org/data/icd9-icd-10-cm-and-pcs-crosswalk-general-equivalence-mapping.html
Note, the latter four datasets are described in more detail in the Supplementary Methods.

Field-specific reporting

Please select the one below that is the best fit for your research. If you are not sure, read the appropriate sections before making your selection.

☒ Life sciences ☐ Behavioural & social sciences ☐ Ecological, evolutionary & environmental sciences

For a reference copy of the document with all sections, see nature.com/documents/nr-reporting-summary-flat.pdf

Life sciences study design

All studies must disclose on these points even when the disclosure is negative.

Sample size

Two datasets were selected to perform the analyses described in this manuscript: the UK Biobank (N=502,048) and the UCSF Clinical Data Warehouse (N=1,204,212). The former dataset was a priori selected because it uniquely contains the information required to conduct this study (structured clinical data, common variant genotypes, and exome sequencing). The UCSF Clinical Data Warehouse was incorporated into the analysis to ensure that the cryptic phenotypes isolated from the UK Biobank generalized to another clinical dataset with distinct ascertainment, demographics and provenance. Using these two datasets, we were able to isolate and replicate Mendelian disease cryptic phenotypes, which were then used to identify common variation associated with Mendelian disease severity. Ideally, our results would have been replicated in an independent genetic dataset, but the UK Biobank was the only publicly available dataset that contained the information required (structured clinical data, common variant genotypes, and exome sequencing) at the time that this study was performed.

Data exclusions

Individual participants were excluded from the UK Biobank analyses according to recommended best practices. The details concerning our exclusion criteria are provided in the Supplementary Methods. These criteria have been previously published (see https://www.nature.com/articles/s41586-018-0579-z and Supplementary Methods) and were decided upon before conducting the analyses reported in the study.

Replication

Complete replication of our findings in an orthogonal dataset was not possible due to limited data availability. Analyses that we conducted to validate our results are described in the main text, which include assessing clinical outcomes in withheld Mendelian disease patients.

Randomization

This is a cohort analysis, so randomization was unnecessary. That said, we did select random subsets of the UCSF and UKBB datasets for model inference and validation. Details regarding this randomization are provided in the text. In addition, given that our analyses were not fully randomized, multiple covariates were incorporated. These include sex, age, genotyping platform (where applicable), smoking history

	(where applicable), and genetic ancestry (using the first 10 principal components of the genetic relationship matrix). Additional details are provided in the Methods section of the main text.
Blinding	There were no treatments, so blinding was unnecessary.

Reporting for specific materials, systems and methods

We require information from authors about some types of materials, experimental systems and methods used in many studies. Here, indicate whether each material, system or method listed is relevant to your study. If you are not sure if a list item applies to your research, read the appropriate section before selecting a response.

Materials & experimental systems

n/a	Involved in the study
☒	☐ Antibodies
☒	☐ Eukaryotic cell lines
☒	☐ Palaeontology and archaeology
☒	☐ Animals and other organisms
☐	☒ Human research participants
☒	☐ Clinical data
☒	☐ Dual use research of concern

Methods

n/a	Involved in the study
☒	☐ ChIP-seq
☒	☐ Flow cytometry
☒	☐ MRI-based neuroimaging

Human research participants

Policy information about studies involving human research participants

Population characteristics

The UCSF Clinical Data Warehouse is a convenience sample of de-identified medical records for all patients who sought care at the UCSF Medical Center between 2012 and 2019 (with some legacy data dating back to 1989). In total, the dataset (at the time of acquisition) contained 1,204,212 unique, de-identified subjects. The demographics of this dataset reflect the patient population that was treated at the medical center during this time frame, which includes all ages (range: 0 to >100), both sexes (46% male; 54% female), and a variety of ethnic/ancestral backgrounds (64% White; 12% Hispanic or Latino, 24% Other). Our final, filtered version of the UCSF dataset (see Supplementary Methods) contained clinical diagnostic information derived from 10,483 unique ICD10-CM codes. These were in turn translated into 1,674 HPO terms. If using the UKBB-ICD10 encoding, the UCSF dataset contained diagnostic information derived from 4,932 ICD10 and 1,423 HPO terms. In addition, diagnostic information was available for 166 unique Mendelian diseases, 50 of which had a prevalence greater than (or equal to) 1:100,000. Additional information regarding dataset filtering, processing, and ICD10-to-HPO alignment can be found in the Methods and Supplementary Methods.

The details concerning the demographic makeup of the UK Biobank have been published previously: https://www.nature.com/articles/s41586-018-0579-z. Briefly, our subset of the UKBB (after removing withdrawn subjects) contained 502,488 unique subjects. The demographics of this dataset reflect the recruitment strategy for the UKBB. Ages (at the time of our initial analyses) ranged from 49-86 yo, with approximately 46% identifying as male. Due to the availability of genetic information (common variant SNP genotypes available for 484,997 subjects after quality control, see Methods/Supplementary Methods), ancestral background could be estimated using a combination of self-reported ethnicity and genotypic information. Our analyses estimate that approximately 84% of the UKBB has a European/Caucasian ancestral background. Similar to the UCSF dataset (when using the UKBB encoding), the UKBB dataset contains diagnostic information derived from 4,932 ICD10 and 1,423 HPO terms (after filtering). Due to it's less granular encoding, only 89 unique Mendelian conditions had diagnostic information available in the UKBB. Importantly, the UKBB is unique in that it has common variant genotypic information and exome sequencing data available for a substantial number of participants (484,997 and 199,234 respectively after quality-control filtering, see Methods/Supplementary Methods).

Recruitment

The UCSF Clinical Data Warehouse is a convenience sample of patients who sought care at the UCSF Medical Center between 2012 and 2019 (with some legacy data dating back to 1989). As a result, this cohort may not be representative of the general population. Additional information regarding the demographics for the UCSF dataset is provided above. The recruitment procedures for the UK Biobank participants have been described in detail elsewhere: https://www.ncbi.nlm.nih.gov/pmc/articles/PMC4380465/.

Ethics oversight

UCSF Institutional Review Board (IRB #: 19-29458)

Note that full information on the approval of the study protocol must also be provided in the manuscript.
